# Supplementary material for: The impact of management compliance attention and board faultlines strength on ESG performance: Evidence from Chinese listed companies
Source: PLoS One. 2025 Aug 22;20(8):e0331004. doi: 10.1371/journal.pone.0331004 (PMC12373182; doi:10.1371/journal.pone.0331004)
Supplement: Attachment — Submitted filename: Comments for Authors.docx [file pone.0331004.s002.docx]

Dear Editorial Manager - PLOS ONE

Thank you for providing me with an opportunity to review the paper titled *"Management compliance attention, board faultlines strength and ESG performance:*

*Evidence from China".*

I would like to offer the following suggestions for the author(s) as a major revision.

| 1 | **Suggested Tile:** The Impact of Management Compliance Attention and Board Faultlines Strength on ESG Performance: Evidence from Chinese Listed Companies |
| --- | --- |
| 2 | **Abstract –** Rewrite the abstract as:   a. Begin with a clear statement of research purpose that links the concept of compliance attention to ESG performance. b. For methods part, add a brief reference to empirical approach or analytical techniques used. c. Emphasize findings with clearer cause-effect language and a more academic tone. Also, moderations and mediation. Use a more structured phrasing to emphasize the mediating role. d. Make the theoretical and practical implications more explicit for common readers. |
| 2 | **Introduction -**   a. Begin with a broader context and relevance of ESG performance. Use updated and sources of citations (not older than 4 years). b. Highlight the gap between global ESG trends and Chinese firms’ practices. c. Currently, research questions are implied. Make them explicit and aligned with your variables. d. Some citations are outdated or less prominent. e. se active voice and precise academic language. Avoid redundancy and informal phrasing (e.g., “company first needs to…” or “frustration of many transnational companies…”). |
| 3 | **Literature Review and Hypotheses Development -** Your literature review shows a promising foundation, drawing on relevant studies and theories. However, it can be significantly improved in structure, clarity, theoretical integration, and academic depth. Below are detailed suggestions across four key dimensions: structure, theoretical underpinning, literature currency, and writing quality.   a. Add brief introductions to each subsection explaining why the topic is relevant to the study. [2.1 Management Compliance Attention and ESG Performance, 2.2 Board Faultlines Strength and ESG Performance, 2.3 Moderating Role of Board Faultlines in the Compliance–ESG Link,] b. Currently used theories (e.g., Compliance Theory, Cognitive Theory, Upper Echelons Theory, and Legitimacy Theory) are appropriate, but they need deeper integration and explanation. c. Cognitive Theory - Connect more directly with managerial perception and ESG commitment. Explain how cognitive framing influences resource allocation to ESG. d. Upper Echelons Theory - Integrate more robustly with board faultlines. Show how board members’ characteristics shape strategic choices like ESG investments. e. Read - Liu, Q., Tang, Y., & Zhang, Y. (2022). ESG performance and firm value: Evidence from China's A-share listed companies. Sustainability, 14(4), 2105. f. Need more clarity, for example - **Original:** Female directors generally have a positive influence on ESG disclosure quality, except for the governance component. **Improved:**While female directors typically enhance the quality of ESG disclosures, their impact on governance-related disclosures may vary (Wasiuzzaman & Subramaniam, 2023). |
| 4 | **Methodology** – Following points need to address:   a. Justify Time Frame: Explain why 2010–2022 was chosen. Was it due to data availability, policy relevance (e.g., ESG regulations in China), or economic events? b. Address Survivorship Bias- Mention whether you account for firms that were delisted or suspended during the sample period. This may skew ESG performance results. c. Specify whether certain industries are overrepresented or underrepresented after exclusion and how that may affect generalizability. d. Clarify "Abnormal Data": e. Explain why the natural logarithm is used (e.g., to normalize skewed distribution, reduce heteroscedasticity). f. Explain how each control variable is theoretically linked to ESG performance (e.g., firm size via number of employees, leverage’s effect on CSR behavior). g. The mediating variable "Orgre" is mentioned in the table but not explained in the main text. Clarify its theoretical basis and how it's tested. |
| 5 | **Data Analysis and Findings** –  My suggestions are here:   a. Explain what an ESG score of 3.202 or a Com_att value of 1.980 practically means. E.g., does 3.2 on ESG imply moderate performance? b. Ensure variable names in tables are intuitive (e.g., use “Compliance Attention” instead of “Com_att”). c. You report statistical significance, but what does a 0.041 increase in ESG performance imply in real terms? Add interpretation of effect size. d. For model fitness - Report R-squared and F-statistics to evaluate how well your model explains the variation in ESG. e. Clarify Column Labeling: For readability, explicitly name models in the narrative (e.g., “Model 4” instead of “M4”). f. Justify Variable Substitution: Why was “law” removed for the Comp_attrb robustness check? Add rationale. Summarize Patterns: Briefly summarize how results from M1–M3 confirm the original findings (e.g., “All variations consistently support H1–H3”). g. Consider using bootstrapping for mediation instead of (or in addition to) the Sobel test for more robust inference. Clarify Z-value Magnitude: Explain what Z = 3.089 means in lay terms — it might not be intuitive to all readers. |
| 6 | **Conclusion and Implications – authors are suggested:**   a. Separate “Conclusions” from “Implications”: This improves clarity, especially for academic and policy-oriented readers. Suggested headers: 6. Conclusions, 7. Practical and Theoretical Implications b. Conclusions - Be concise and focused. Avoid repeating long discussions from the results section. Highlight the contribution to theory. E.g., how the study adds to ESG or governance literature. Group findings logically. Use phrases like “First, Second, Third” more effectively. **Example:** This study investigates how management compliance attention affects ESG performance, using A-share listed companies from 2010 to 2022. Based on extensive empirical analysis, the following key conclusions are drawn…………………………… c. Implications - Separate practical from theoretical implications. Make implications actionable. Specify what, who, and why. Avoid repetition of earlier findings; focus on what stakeholders should do. d. Add a short paragraph to conclude both sections with a forward-looking statement (future directions), e.g. Future research could explore other organizational factors, such as corporate culture or innovation orientation, that may further explain how firms can sustainably manage ESG responsibilities in dynamic environments………………….. |
|  | This paper has a scope but still needs major improvement with revisions as suggested above. |
