# Supplementary material for: The impact of management compliance attention and board faultlines strength on ESG performance: Evidence from Chinese listed companies
Source: PLoS One. 2025 Aug 22;20(8):e0331004. doi: 10.1371/journal.pone.0331004 (PMC12373182; doi:10.1371/journal.pone.0331004)
Supplement: Attachment — Submitted filename: Response to Reviewers.docx [file pone.0331004.s005.docx]

**Response to editors**

**1.** ***The reviewers found that the manuscript references multiple theories—such as stakeholder theory, upper echelons theory, compliance theory, and dynamic capabilities theory—but lacks proper integration of these frameworks into the hypotheses and interpretations. Moreover, central constructs such as management compliance attention, board faultlines strength, and organizational resilience are insufficiently defined and under-theorized. A more structured and theory-driven conceptual model is essential.***

**Response:**

The manuscript now systematically integrates theories into hypothesis development, with clear construct definitions and a theory-driven conceptual model:

(1) Hypothesis 1 is anchored in Institutional Theory, positing that management compliance attention drives ESG performance by aligning with regulatory/normative pressures. The construct "management compliance attention" is defined as managerial focus on regulatory requirements, operationalized via text-mining of annual reports (e.g., keyword frequency for "compliance," "regulations") and natural logarithm transformation to reduce heteroscedasticity.
(2) Hypothesis 2 leverages Upper Echelons Theory to link board faultlines strength to ESG performance, defining "board faultlines strength" as structural divisions within boards based on demographic attributes (gender, age, education, tenure), measured using the FLS index (Lau & Murnighan, 1998).
(3) Hypothesis 3 integrates both theories, demonstrating how board faultlines moderate the compliance-ESG relationship.
(4) The mediating role of "organizational resilience" is rooted in Dynamic Capabilities Theory, defined as adaptive capacity and measured via three-year sales growth and stock return volatility (entropy weight method).

Figure 1 illustrates the pathway: "Institutional Identification → Capability Building → Performance Enhancement", ensuring alignment between constructs and theories.

**2.*While the manuscript cites a wide range of prior studies, the literature review lacks coherence and does not effectively build a critical narrative or justify the research gap. Furthermore, the manuscript should better*** ***articulate its theoretical and practical novelty compared to existing ESG literature.***

**Response:**

The literature review is restructured to build a critical narrative and justify research gap:

(1) Theoretical coherence. We critiques prior ESG studies for overlooking the interaction between compliance attention and board dynamics, and integrates Institutional Theory with Upper Echelons Theory to explain how regulatory compliance (macro) and board heterogeneity (micro) synergistically impact ESG.
(2) Research gap. Existing ESG literature lacks exploration of the synergistic effects between management compliance attention and board structure, particularly in transitional economies, failing to reveal how board faultlines moderate the translation of compliance into ESG performance. Additionally, the mediating role of organizational resilience in this relationship remains underexplored, and most studies overlook heterogeneous impacts of ownership and market competition, limiting theoretical applicability to China’s institutional context.
(3) Theoretical novelty. Integrating Institutional Theory, Upper Echelons Theory, and Dynamic Capabilities Theory, this study constructs a theoretical model of "compliance attention-board faultlines-organizational resilience-ESG performance". It demonstrates that board faultlines amplify the positive effect of compliance attention through enhanced supervision and strategic innovation, and identifies organizational resilience as a mediator in this pathway. For the first time in transitional economies, it reveals the interaction between formal institutions (policy pressures) and informal governance (board diversity), expanding the theoretical boundaries of ESG governance.
(4) Practical novelty. This study proposes differentiated ESG strategies: state-owned enterprises should leverage policy resources to drive the compliance-ESG chain, while private firms need to activate compliance effectiveness via board diversity. Firms in low-competition industries are advised to build reputational capital through proactive compliance. It suggests regulators enhance ESG disclosure standards and enterprises strengthen compliance cultures and board diversity, providing an operational framework for corporate governance and sustainable development in emerging markets.

**3. *Reviewers flagged numerous grammatical, syntactical, and stylistic issues that hinder clarity and diminish the overall academic tone. A thorough English language revision, preferably by a professional editor or native speaker, is strongly recommended to enhance readability and scholarly presentation.***

**Response:**

(1) Correct grammatical error. We ensure tense consistency, subject-verb agreement, and proper preposition usage.
(2) Enhance academic tone. We Replaced informal phrasing with precise terminology.
(3) Improve clarity. We standardized the variable names, described the methods in the active voice, and made the statements more logical.

**4.** ***The current data availability statement ("available on request") does not meet PLOS ONE’s open data policy. The authors must either deposit the data in a public repository or clearly explain any restrictions along with justified access procedures.***

**Response:**

In alignment with PLOS ONE’s open data policy:

(1) Data deposit: The dataset, processing code, and empirical results are uploaded as supporting files.

(2) Availability statement: The manuscript now states: "All relevant data are within the manuscript and its Supporting Information files".

(3) Accessibility: No restrictions apply; public reuse is permitted with proper citation.

**5.** ***The implications of the findings for corporate governance, managerial behavior, and ESG policy are currently underdeveloped. Additionally, the limitations section should address the scope of generalizability, especially given the context-specific nature of Chinese listed firms and the use of text-mining techniques.***

**Response:**

(1) Corporate governance implications.

①State-owned companies. Leverage policy mandates to lead in ESG via institutional resources.

②Private companies. Prioritize board diversity to compensate for weak regulatory pressure.

(2) ESG policy implications. Regulators should enhance disclosure standards and integrate compliance frameworks with sustainability goals.
(3) Limitations:

①Generalizability. Sample specificity may not apply to non-transitional economies or non-listed firms.

②Text-mining biases. Keyword selection may overlook implicit compliance practices; future studies could triangulate management compliance attention with qualitative data (e.g., management interviews) or behavioral indicators (e.g., regulatory violation records) .

(4) Future directions. Explore industry-specific effects, regional disparities in China, or cultural factors as moderators of ESG performance.

**Response to the Review Comments from the First Reviewer**

**1.*The definition and measurement of key constructs like "management compliance attention" are not clear enough. The method of using keyword frequency from annual reports is interesting, but the selection of keywords lacks theoretical support, and the approach should be explained more carefully to allow replication. Similarly, the calculation of board faultlines strength is mathematically complex, but conceptually not well introduced. Readers without technical expertise may find it difficult to understand what the metric represents and why it matter***

**Response:**

We supplemented conceptual definitions and theoretical justifications for "management compliance attention" and "board faultlines strength" and provided a detailed explanation of the variable construction process for both.

**2. *While the statistical techniques used—including regression analysis, interaction terms, and robustness tests—are appropriate, the paper does not provide sufficient explanation for the use of lagged variables as instruments in the 2SLS regression. The authors should clarify why these instruments are valid and how the assumptions behind them are satisfied.***

**Response:**

We added an explanation in the text about the rationality and validity of using lagged variables as instrumental variables in the 2SLS regression.

**3.** ***There are concerns regarding data availability. The authors use proprietary data from the CSMAR and CNRDS databases. Although this is common in research, the paper should include more detailed information on how others can access the data and replicate the key variables, especially those created through text analysis.***

**Response:**

We provided a more detailed explanation of the data acquisition and construction process for the variables "Management compliance attention" and "Board faultlines strength".

**4.** ***The language needs substantial revision. There are many grammatical errors, awkward phrases, and unclear sentences that affect readability throughout the paper. This is especially noticeable in the abstract, introduction, and results sections. The manuscript would benefit from thorough editing by a native English speaker or professional language editor.***

**Response:**

We corrected grammatical errors and improved language expression throughout the manuscript as much as possible.

**5. *The literature review is too broad and lacks clear structure. While the authors cite many relevant studies, they need to organize the section more logically and connect the literature more directly to their hypotheses. The theoretical framework (e.g., stakeholder theory and upper echelons theory) is mentioned but not fully developed or integrated into the study.***

**Response:**

We reorganized the theoretical basis for the research hypotheses, supporting Hypothesis 1 with "Institutional Theory," Hypothesis 2 with "Upper Echelons Theory," Hypothesis 3 with an integration of "Institutional Theory" and "Upper Echelons Theory," and the mediating effect with "Dynamic Capabilities Theory." We rewrote Sections 2.1 and 2.2 to more directly link the literature with the hypotheses and integrated the theoretical analysis.

**6. *Although the research contributes new insight, the authors should more clearly state what is unique about their study compared to existing work. Explaining the novelty in a few sentences will make the paper’s contribution stand out more.***

**Response:**

In the last paragraph of Section "1. Introduction", we re-summarized the paper’s contributions, clarified its research innovations, and added Section "7. Theoretical Implications" to elaborate on extensions and contributions to existing theories.

**7. *The practical implications are currently underdeveloped. The authors briefly mention that firms should enhance compliance and board diversity, but they should expand on this. What specific steps should firms, managers, or policymakers take based on these findings?***

**Response:**

We added Section "8. Practical implications" to elaborate on practical implications and recommendations, and Section "9. Limitations and Future Research" to address existing issues and future research directions.

**Response to the Review Comments from the Second Reviewer**

| 1 | **Suggested Tile:** The Impact of Management Compliance Attention and Board Faultlines Strength on ESG Performance: Evidence from Chinese Listed Companies  **Response:**  We have revised the title as suggested. |
| --- | --- |
| 2 | **Abstract –** Rewrite the abstract as:   a. Begin with a clear statement of research purpose that links the concept of compliance attention to ESG performance.   **Response:**  We newly added and clarified research objective: this study endeavors to empirically examine the impact of management compliance attention and board faultlines strength on ESG performance by analyzing a sample from Chinese A-share listed companies spanning 2010 to 2022.   b. For methods part, add a brief reference to empirical approach or analytical techniques used.   **Response:**  We newly added and clarified research method: By leveraging textual analysis of annual reports to construct the variable of managerial attention to compliance, we adopt the OLS (Ordinary Least Squares) regression model along with moderation and mediation analyses for empirical testing.   c. Emphasize findings with clearer cause-effect language and a more academic tone. Also, moderations and mediation. Use a more structured phrasing to emphasize the mediating role.   **Response:**  We used clearer cause-effect expressions and academic language to highlight the findings including the moderation and mediation effects: The study finds three key results: (1) Management compliance attention is positively correlated with ESG performance (β=0.041, p<0.01); (2) Board faultlines strength significantly strengthens the positive impact of management compliance attention on ESG performance (β=0.114, p<0.01); (3) Organizational resilience partially mediates the relationship between management compliance attention and ESG performance, as indicated by a Sobel test (z=3.089, p=0.002).   d. Make the theoretical and practical implications more explicit for common readers.   **Response:**  We refined theoretical and practical implications: The findings contribute to the literature by integrating institutional theory, upper echelons theory, and dynamic capabilities theory, demonstrating that management compliance attention enhances ESG performance through the cultivation of organizational resilience, with board faultlines serving as a critical moderator. Practical implications include recommendations for firms to strengthen compliance cultures, cultivate diverse boards, and build organizational resilience. Policymakers and regulators are urged to enhance compliance frameworks and ESG disclosure standards to foster sustainable corporate practices. |
| 2 | **Introduction -**   a. Begin with a broader context and relevance of ESG performance. Use updated and sources of citations (not older than 4 years).   **Response:**  Except for retaining some classic theoretical literature (such as Agency Cost Theory (Jensen & Meckling, 1976); Upper Echelons Theory (Hambrick & Mason, 1984), we updated the vast majority of the literature to the recent four years.   b. Highlight the gap between global ESG trends and Chinese firms’ practices.   **Response:**  We cited literature indicating the scale of global ESG investment and the importance of ESG performance, and pointed out the heterogeneity and particularity of ESG performance of Chinese enterprises.   c. Currently, research questions are implied. Make them explicit and aligned with your variables.   **Response:**  In the penultimate paragraph of the introduction, we raised the research questions explicitly, pointed to key variables such as management compliance attention, ESG performance, and board faultlines strength.   d. Some citations are outdated or less prominent.   **Response:**  We cited literature in recent years(not older than 4 years) ,which are highly relevant to the research topic.   e. se active voice and precise academic language. Avoid redundancy and informal phrasing (e.g., “company first needs to…” or “frustration of many transnational companies…”).   **Response:**  We have organized sentences in active voice and precise academic language. |
| 3 | **Literature Review and Hypotheses Development -** Your literature review shows a promising foundation, drawing on relevant studies and theories. However, it can be significantly improved in structure, clarity, theoretical integration, and academic depth. Below are detailed suggestions across four key dimensions: structure, theoretical underpinning, literature currency, and writing quality.   a. Add brief introductions to each subsection explaining why the topic is relevant to the study. [2.1 Management Compliance Attention and ESG Performance, 2.2 Board Faultlines Strength and ESG Performance, 2.3 Moderating Role of Board Faultlines in the Compliance–ESG Link,]   **Response:**  We added a brief introduction to the beginning of each subsection (2.1-2.3) explaining the relevance of the topic to this study.   b. Currently used theories (e.g., Compliance Theory, Cognitive Theory, Upper Echelons Theory, and Legitimacy Theory) are appropriate, but they need deeper integration and explanation.   **Response:**  We reconstructed and integrated Institutional Theory and Upper Echelons Theory.   c. Cognitive Theory - Connect more directly with managerial perception and ESG commitment. Explain how cognitive framing influences resource allocation to ESG.   **Response:**  We re-integrated the original 'Cognitive Theory' into Institutional Theory, and systematically discussed the impact of management compliance attention on ESG performance together with 'regulatory' and 'normative' dimensions.   d. Upper Echelons Theory - Integrate more robustly with board faultlines. Show how board members’ characteristics shape strategic choices like ESG investments.   **Response:**  We re-wrote '2.2 Board faultlines strength and ESG performance' to more closely integrate Upper Echelons Theory with board faultlines strength and clearly explain the impact of board faultlines strength on ESG performance.   e. Read - Liu, Q., Tang, Y., & Zhang, Y. (2022). ESG performance and firm value: Evidence from China's A-share listed companies. Sustainability, 14(4), 2105.   **Response:**  Unfortunately, we were unable to locate this literature through known channels such as Google Scholar and the Sustainability journal. If possible, could you please send us this paper? We will read it carefully and explore its connection with our study.   f. Need more clarity, for example - **Original:** Female directors generally have a positive influence on ESG disclosure quality, except for the governance component. **Improved:**While female directors typically enhance the quality of ESG disclosures, their impact on governance-related disclosures may vary (Wasiuzzaman & Subramaniam, 2023).   **Response:**  We improved the expression throughout the text as much as possible to make it clearer. |
| 4 | **Methodology** – Following points need to address:   a. Justify Time Frame: Explain why 2010–2022 was chosen. Was it due to data availability, policy relevance (e.g., ESG regulations in China), or economic events?   **Response:**  We added a textual explanation in "3.1 Data collection" to explain the reasons for selecting the sample period of 2010-2022.   b. Address Survivorship Bias- Mention whether you account for firms that were delisted or suspended during the sample period. This may skew ESG performance results.   **Response:**  We added a textual explanation in "3.1 Data collection": Samples of delisted or suspended companies were excluded.   c. Specify whether certain industries are overrepresented or underrepresented after exclusion and how that may affect generalizability.   **Response:**  We added a textual explanation in "3.1 Data collection" to explain the reasons for excluding samples of financial companies, ST and *ST companies, and conducted the analysis of the remaining company samples.   d. Clarify "Abnormal Data":   **Response:**  In the original text, extreme values at the 1st and 99th percentiles were defined as "Abnormal Data", and such extreme values had already been excluded during data processing, so there is no "Abnormal Data" left. We added a textual statement "Additionally, we excluded extreme values at the 1st and 99th percentiles." in "3.1 Data collection".   e. Explain why the natural logarithm is used (e.g., to normalize skewed distribution, reduce heteroscedasticity).   **Response:**  We added a textual explanation in "3.1 Data collection": To reduce the probability of heteroscedasticity in the linear regression model, we applied natural logarithm transformations to the values of the dependent variable, independent variable, and some control variables.   f. Explain how each control variable is theoretically linked to ESG performance (e.g., firm size via number of employees, leverage’s effect on CSR behavior).   **Response:**  We explained the theoretical connections between each control variable and ESG performance in "3.2.4 Control variables".   g. The mediating variable "Orgre" is mentioned in the table but not explained in the main text. Clarify its theoretical basis and how it's tested.   **Response:**  In "5.5 The mediating role of organizational resilience", We re-cited literature to elaborate on the concept, theoretical basis, and variable construction method of "Orgre". |
| 5 | **Data Analysis and Findings** –  My suggestions are here:   a. Explain what an ESG score of 3.202 or a Com_att value of 1.980 practically means. E.g., does 3.2 on ESG imply moderate performance?   **Response:**  We re-wrote the content of "4.1 Descriptive statistics" to present the means and standard deviations of Compliance Attention and ESG performance before natural logarithm transformation, and explained the practical meanings of these two variables' data.   b. Ensure variable names in tables are intuitive (e.g., use “Compliance Attention” instead of “Com_att”).   **Response:**  We used "Compliance Attention" instead of "Com_att" and "Board Faultlines" instead of "Mfls" to make variable names clearer and more understandable.   c. You report statistical significance, but what does a 0.041 increase in ESG performance imply in real terms? Add interpretation of effect size.   **Response:**  We supplemented and improved the practical implications of the impact of compliance attention on ESG performance in "4.3 Regression analysis".   d. For model fitness - Report R-squared and F-statistics to evaluate how well your model explains the variation in ESG.   **Response:**  We reported R-squared and F-statistics in "4.3 Regression analysis" to evaluate how well the model explains the variation in ESG.   e. Clarify Column Labeling: For readability, explicitly name models in the narrative (e.g., “Model 4” instead of “M4”).   **Response:**  We clarified model names by replacing "M" with "Model" in the text.   f. Justify Variable Substitution: Why was “law” removed for the Comp_attrb robustness check? Add rationale. Summarize Patterns: Briefly summarize how results from M1–M3 confirm the original findings (e.g., “All variations consistently support H1–H3”).   **Response:**  We re-wrote the paragraph on replacing the independent variable in "5.2 Replacement of variables" to explain why the keyword 'law' was excluded and re-explained the regression results; added a final paragraph in "4.3 Regression analysis" to summarize the results after variable substitution.   g. Consider using bootstrapping for mediation instead of (or in addition to) the Sobel test for more robust inference. Clarify Z-value Magnitude: Explain what Z = 3.089 means in lay terms — it might not be intuitive to all readers.   **Response:**  In the last paragraph of "5.5 The mediating role of organizational resilience", we used bootstrapping for mediation, results show the mediating effect of organizational resilience is significant. Additionally we added a textual explanation to clarify the meaning and practical significance of the Z-value. |
| 6 | **Conclusion and Implications – authors are suggested:**   a. Separate “Conclusions” from “Implications”: This improves clarity, especially for academic and policy-oriented readers. Suggested headers: 6. Conclusions, 7. Practical and Theoretical Implications   **Response:**  We added "6. Conclusions" to elaborate on the conclusions. Additionally, to clarify theoretical and practical implications more clearly, "7. Theoretical Implications" and "8. Practical Implications" were separately added.   b. Conclusions - Be concise and focused. Avoid repeating long discussions from the results section. Highlight the contribution to theory. E.g., how the study adds to ESG or governance literature. Group findings logically. Use phrases like “First, Second, Third” more effectively. **Example:** This study investigates how management compliance attention affects ESG performance, using A-share listed companies from 2010 to 2022. Based on extensive empirical analysis, the following key conclusions are drawn……………………………   **Response:**  In "6. Conclusions", the main conclusions were presented in a list, we highlighted the study's contribution to theoretical development.   c. Implications - Separate practical from theoretical implications. Make implications actionable. Specify what, who, and why. Avoid repetition of earlier findings; focus on what stakeholders should do.   **Response:**  We elaborated on theoretical and practical implications separately, and provided relevant recommendations for stakeholders in "8. Practical Implications".   d. Add a short paragraph to conclude both sections with a forward-looking statement (future directions), e.g. Future research could explore other organizational factors, such as corporate culture or innovation orientation, that may further explain how firms can sustainably manage ESG responsibilities in dynamic environments…………………..   **Response:**  We added "9. Limitations and future research" to elaborate on research limitations and future research directions. |

**Response to the Review Comments from the Third Reviewer**

**1.** ***Theoretical Framework Needs Strengthening:***

***The manuscript references several theories (compliance theory, upper echelons theory, cognitive theory, legitimacy theory, dynamic capabilities theory), but these are not well-integrated into the research design.***

***Theoretical frameworks should guide hypothesis development and interpretation of results more clearly.***

***A conceptual model or figure showing the theoretical logic and relationships between variables is recommended.***

**Response:**

We reorganized the theoretical basis for the research hypotheses, supporting Hypothesis 1 with "Institutional Theory", Hypothesis 2 with "Upper Echelons Theory", Hypothesis 3 with an integration of "Institutional Theory" and "Upper Echelons Theory", and the mediating effect with "Dynamic Capabilities Theory". We rewrote Sections 2.1 and 2.2 to integrate theoretical analysis with the research design and drew a logical diagram illustrating how Management Compliance Attention influences ESG performance.

**2.** ***Construct Clarity and Conceptual Definitions:***

***Core constructs such as "management compliance attention," "board faultlines strength," and "organizational resilience" require clearer, theory-driven definitions.***

***Compliance attention is treated as a variable but lacks explanation regarding its strategic or operational implications.***

***The mediating role of organizational resilience is interesting but needs more elaboration in both measurement and conceptual justification.***

**Response:**

We supplemented conceptual definitions and theoretical justifications for "management compliance attention" and "board faultlines strength", provided a detailed explanation of their variable construction processes, and added theoretical explanations for "organizational resilience" and its mediating role.

**3. *Language and Readability:***

***The manuscript contains frequent grammatical errors, awkward phrasing, and inconsistent terminology.***

***Examples include incorrect punctuation (e.g., spaces before commas), subject-verb disagreement, and non-standard academic expressions.***

***A professional English language edit is essential to ensure clarity and professionalism.***

**Response:**

We polished the entire manuscript to minimize formatting and grammatical errors and ensure standardized academic expression.

**4. *Data Availability Policy Not Met:***

***The current data availability statement (“available on request”) does not comply with PLOS ONE’s open data policy.***

***Authors must deposit data in a public repository or justify restrictions with clear access instructions.***

**Response:**

We reuploaded the dataset, dataset-processing code and empirical results files as supporting information files, and added the following statement after the main text: Data availability statement: All relevant data are within the manuscript and its Supporting Information files.

**5. *Literature Review Lacks Coherence:***

***Although many studies are cited, the review lacks synthesis and a critical narrative.***

***The literature should be used to build a clear research gap and support theoretical framing more effectively.***

**Response:**

We reorganized the literature review to more closely link it with theoretical analysis and research hypotheses. In Section 2.1, we supplemented a critical review of the literature to identify research gap and analyzed the positive impact of Management Compliance Attention on ESG performance based on existing literature.

**6. *Mediation and Moderation Analysis Are Valuable:***

***The inclusion of mediation (organizational resilience) and moderation (board faultlines) adds depth to the analysis.***

***However, further discussion is needed to tie these results back to the theoretical context.***

**Response:**

We rewrote Section 4.3 to analyze the empirical results in conjunction with the theoretical framework. In Section 5.5, we not only supplemented the theoretical basis and variable construction of organizational resilience but also discussed the mediating effect results in depth based on theoretical foundations.

**7. *Heterogeneity Analysis Adds Insight:***

***Subgroup comparisons (e.g., high vs. low competition, state vs. private firms) are helpful and relevant.***

***These findings could be more strongly discussed in terms of policy implications.***

**Response:**

We introduce new sections "7.4 Contributing to Transitional Economy Research through Institutional Context Variations" and "8.4 Tailoring Strategies to Ownership and Competitive Contexts", which elaborate on the theoretical implications of heterogeneity analysis and practical recommendations.
