# Supplementary material for: The impact of management compliance attention and board faultlines strength on ESG performance: Evidence from Chinese listed companies
Source: PLoS One. 2025 Aug 22;20(8):e0331004. doi: 10.1371/journal.pone.0331004 (PMC12373182; doi:10.1371/journal.pone.0331004)
Supplement: Attachment — Submitted filename: Second Round - Changes suggested to author.docx [file pone.0331004.s004.docx]

Dear Editorial Manager - PLOS ONE

Thank you for providing me with an opportunity to review the paper titled *"* *The Impact of Management Compliance Attention and Board Faultlines Strength on ESG Performance: Evidence from Chinese Listed Companies".*

**Purpose:** To document reviewer suggestions that remain unaddressed or insufficiently addressed in the revised manuscript.

While the author has addressed most suggestions commendably, the remaining issues—particularly the lack of theoretical integration clarity, missing reference, and incomplete robustness explanation—warrant a minor revision before acceptance.

|  | **Suggestions** | **Unmet** |
| --- | --- | --- |
| 1 | Justify variable substitution in robustness check (e.g., removal of "law" keyword) | Not Met No rationale is provided for keyword exclusion. Robustness substitution logic remains unclear. |
| 2 | Deeper integration of Cognitive Theory | Partially Met Cognitive framing is vaguely tied under Institutional Theory; clarity on ESG implications is lacking. |
| 3 | Interpret effect size of regression coefficients (e.g., β = 0.041) | Partially Met Still lacks plain-language explanation of practical significance of the coefficients. |
| 4 | Explain Z = 3.089 result and provide bootstrapping analysis | Partially Met Bootstrapping was mentioned, but without numerical outputs or confidence intervals. |
| 5 | Link findings more explicitly to the theoretical framework in the Discussion section | Partially Met Theories are referenced, but empirical-to-theoretical discussion is not well-integrated. |
| 6 | Improve grammar, readability, and academic tone | Partially Met Language improved overall, but several long, awkward, or informal phrasings still remain. |
|  |  |  |
|  |  |  |
