# Supplementary material for: The impact of management compliance attention and board faultlines strength on ESG performance: Evidence from Chinese listed companies
Source: PLoS One. 2025 Aug 22;20(8):e0331004. doi: 10.1371/journal.pone.0331004 (PMC12373182; doi:10.1371/journal.pone.0331004)
Supplement: Attachment — Submitted filename: Second_Round_-_Changes_suggested_to_author_auresp_2.docx [file pone.0331004.s006.docx]

|  | **Suggestions** | **Response** |
| --- | --- | --- |
| 1 | Justify variable substitution in robustness check (e.g., removal of "law" keyword) | *Not Met No rationale is provided for keyword exclusion. Robustness substitution logic remains unclear.*  Response:  In the revised manuscript, we have supplemented the justification for removing the "law" keyword as follows:  1.The keyword "law" primarily represents the foundational legal framework, setting mandatory boundaries for corporate behavior. However, specific compliance practices require enterprises to extend beyond mere legal provisions, integrating institutional pressures across regulative, normative, and cognitive dimensions. Excluding "law" serves two key purposes:  (1)Test dependency on mandatory legal pressures: By removing this strongly symbolic term, we verify whether the observed relationship between management compliance attention and ESG performance relies on passive responses to legal mandates or reflects a more holistic attention to multi-dimensional compliance.  (2)Capture cognitive breadth of compliance attention: "Law" may constrain management focus to narrow legal frameworks, potentially overlooking broader compliance dimensions. Retaining significance after exclusion confirms that compliance attention drives ESG performance through systematic cognition of institutional environments, not just perfunctory mentions of legal terms.  2.We have also clarified the substitution logic for the moderating variable (board faultlines strength).The original and alternative measures (Board Faultlines and NBoard Faultlines) both quantify subgroup divisions but use distinct weighting schemes for demographic attributes (gender, age, education, tenure). This substitution tests whether conclusions depend on specific attribute weightings, ensuring the moderating effect of faultlines is not artifactually tied to a single calculation method. |
| 2 | Deeper integration of Cognitive Theory | *Partially Met Cognitive framing is vaguely tied under Institutional Theory; clarity on ESG implications is lacking.*  Response:  We have integrated institutional theory and cognitive theory， and elaborated on practical implications for ESG, as detailed below:  We explicitly distinguish the complementary roles of institutional theory and cognitive theory in shaping ESG performance.Institutional Theory explains the external impetus: regulative, normative, and cognitive pressures, compelling firms to align with institutional demands.Cognitive Theory illuminates the internal translation mechanism: Management’s compliance attention acts as a critical interface, converting abstract institutional pressures into actionable ESG practices through "interpretation, decomposition, and meaning attribution."  This integration shows that cognitive framing ensures institutional pressures are not just passively adhered to but actively leveraged to improve ESG performance, bridging the gap between external mandates and internal action.These revisions clarify how cognitive processes mediate institutional influences on ESG outcomes, and enhance the practical relevance of our findings. |
| 3 | Interpret effect size of regression coefficients (e.g., β = 0.041) | *Partially Met Still lacks plain-language explanation of practical significance of the coefficients.*  Response:  We have supplemented concrete interpretations of key coefficients across empirical analyses, as below:  1. Baseline Regression (Table 4, Model 4)  The coefficient of management compliance attention (β=0.041, p<0.01) indicates that a one-unit increase in compliance attention is associated with a 0.041-unit increase in the natural logarithm of ESG scores, reflecting that heightened managerial focus on compliance directly enhances performance across environmental, social, and governance dimensions.  The interaction term (β=0.114, p<0.01) means that stronger board faultlines amplify the compliance-ESG relationship: For each unit increase in faultline strength, the marginal effect of compliance attention on ESG performance rises by 0.114. This indicates that diverse boards strengthen the translation of compliance focus into tangible ESG outcomes.  2. Robustness Test with Variable Substitution (Table 5)  After excluding the "law" keyword, the coefficient of adjusted compliance attention (β=0.041, p<0.01) remains identical to the baseline regression, confirming that the relationship is not driven by passive mentions of legal terms but by holistic compliance cognition, not just legal obligations.  3. Heterogeneity Analysis (Table 7)  In low-competition environments, compliance attention has a stronger impact (β=0.047 vs. 0.039 in high-competition settings), meaning one-unit increase in compliance attention is associated with 0.047 unit increase in ESG performance. State-owned enterprises (SOEs) show a larger coefficient (β=0.045 vs. 0.037 for private firms), likely due to stronger alignment with policy-driven institutional pressures. |
| 4 | Explain Z = 3.089 result and provide bootstrapping analysis | *Partially Met Bootstrapping was mentioned, but without numerical outputs or confidence intervals.*  Response:  To further validate the mediating effect, we conducted a bootstrap test with 117 resamplings. The results show that Z-value is 3.24, which exceeds the 2.58 threshold, reinforcing the significance of the mediating effect at the 1% level, and that 95% confidence interval is between 0.0003 and 0.0012 for the indirect effect. Since confidence interval does not include zero, it provides additional statistical evidence that organizational resilience significantly mediates the relationship between management compliance attention and ESG performance. |
| 5 | Link findings more explicitly to the theoretical framework in the Discussion section | *Partially Met Theories are referenced, but empirical-to-theoretical discussion is not well-integrated.*  Response:  We have explicitly linked key results to the four theoretical lenses in the Conclusions section, as detailed below:  1. Institutional Theory & Cognitive Theory Integration. Institutionally, firms gain legitimacy by aligning with regulative and normative pressures, which our findings confirm through compliance attention’s association with improved ESG practices. Cognitively, the robustness of this relationship even after excluding the "law" keyword demonstrates that management translates abstract institutional pressures into strategic action through cognitive frameworks, bridging external mandates and internal execution.  2. Upper Echelons Theory. The moderating role of board faultlines strength supports the theory’s core premise that top management heterogeneity shapes organizational outcomes. Our findings show demographic and experiential diversity among directors amplifies the compliance-ESG link by enhancing supervisory depth and strategic breadth.  3. Dynamic Capabilities Theory. The partial mediation of organizational resilience enriches the theory by identifying compliance attention as a driver of adaptive capabilities. Empirically, compliance attention enhances resilience, which in turn boosts ESG performance. This confirms that compliance systems—far from being passive obligations—build dynamic capabilities that underpin sustainable practices, extending the theory’s application to regulatory compliance contexts.  4. Heterogeneity results highlight how institutional contexts shape theoretical mechanisms. For state-owned enterprises, stronger compliance-ESG effects reflect deeper embedding in institutional environments, while private firms’ reliance on board faultlines underscores upper echelons theory’s relevance in weaker institutional settings. This contextualizes the theories within China’s transitional economy, demonstrating their contingent applicability.  These revisions explicitly map empirical results to theoretical propositions, strengthening the integration of findings with the theoretical framework. |
| 6 | Improve grammar, readability, and academic tone | *Partially Met Language improved overall, but several long, awkward, or informal phrasings still remain.*  Response:  We have reorganized the paper and made every effort to address the pointed-out remaining issues concerning grammar, readability, and academic tone. Specifically, we revised awkward expressions to enhance accuracy, split overly long sentences to improve fluency, and refined informal expressions to conform to academic norms. |
